# SEX DIFFERENCES IN THE LINK BETWEEN DNA METHYLATION–BASED BIOLOGICAL AGING AND HEALTH IN OLDER INDIVIDUALS

**DOI:** 10.1093/geroni/igad104.2493

**Published:** 2023-12-21

**Authors:** Aung Zaw Zaw Phyo, Peter Fransquet, Jo Wrigglesworth, Robyn Woods, Sara Espinoza, Joanne Ryan

**Affiliations:** Monash University, Melbourne, Australia. , Melbourne, Victoria, Australia; Deakin University, Burwood East, Victoria, Australia; Monash University, Melbourne, Victoria, Australia; Monash University, Melbourne, Victoria, Australia; Cedars-Sinai Medical Center, Los Angeles, California, United States; Monash University, Melbourne, Victoria, Australia

## Abstract

It is well-established that women live longer than men, however, sex differences in biological aging have not been consistently reported, and may differ depending on the measure used. This study aimed to determine the correlation between DNA methylation (DNAm) age acceleration (AA) measures, with system-wide frailty-index (FI) aging and brain-predicted age difference (brain-PAD), in an initially healthy population of men and women aged ≥70 years. We additionally explored the extent to which these AA measures were associated with clinical measures, and chronic conditions, separately in men and women. DNAm age (Horvath, Hannum, PhenoAge, GrimAge, and DunedinPACE) were estimated in blood from 560 Australians (women, 50.7%) enrolled in the ASPREE study. FI was a deficit accumulation of 67 items including cognition and morbidities. Brain age was estimated from T1-weighted MRI. Clinical measurements, laboratory tests, and self-reports were used to identify chronic illnesses. FI had the strongest correlation with GrimAA and DunedinPACE (range r: 0.20 to 0.24 in both sexes) but was not associated with HorvathAA. Brain-PAD was not correlated with any biological aging measure. Correlations between AA and clinical measures were more commonly found in women than men and were the strongest for DunedinPACE and clinical measures including grip strength, gait speed, and self-rated health (r: -0.23 to 0.24). AA measures, especially GrimAA and DunedinPACE, were significantly associated with hypertension, diabetes, chronic kidney disease in men, and obesity and depression in women. Our findings highlight the importance of considering sex differences when investigating the link between biological age and clinical measures.

